# Targeting the hallmarks of aging to improve influenza vaccine responses in older adults

**DOI:** 10.1186/s12979-023-00348-6

**Published:** 2023-05-17

**Authors:** Andreia N. Cadar, Dominique E. Martin, Jenna M. Bartley

**Affiliations:** UConn Center On Aging and Department of Immunology, University of Connecticut School of Medicine, Farmington, CT 06030 USA

**Keywords:** Geroscience, Hallmarks of aging, Flu vaccine, Vaccine responses

## Abstract

Age-related declines in immune response pose a challenge in combating diseases later in life. Influenza (flu) infection remains a significant burden on older populations and often results in catastrophic disability in those who survive infection. Despite having vaccines designed specifically for older adults, the burden of flu remains high and overall flu vaccine efficacy remains inadequate in this population. Recent geroscience research has highlighted the utility in targeting biological aging to improve multiple age-related declines. Indeed, the response to vaccination is highly coordinated, and diminished responses in older adults are likely not due to a singular deficit, but rather a multitude of age-related declines. In this review we highlight deficits in the aged vaccine responses and potential geroscience guided approaches to overcome these deficits. More specifically, we propose that alternative vaccine platforms and interventions that target the hallmarks of aging, including inflammation, cellular senescence, microbiome disturbances, and mitochondrial dysfunction, may improve vaccine responses and overall immunological resilience in older adults. Elucidating novel interventions and approaches that enhance immunological protection from vaccination is crucial to minimize the disproportionate effect of flu and other infectious diseases on older adults.

## Burden of influenza infection in older adults

Adults over 65 years of age are more susceptible to infectious diseases, such as influenza (flu), and have increased risk for severe illness. During the 2019–2020 flu season, it was estimated that 44% of hospitalizations and 68% of flu-related deaths occurred among older adults [1]. Unfortunately, following laboratory-confirmed flu infections, older adults also commonly have declines in activities of daily living (ADLs) such as bathing, dressing, and walking. In fact, when measuring recovery by measures of ADL, only about 30% of individuals recover to their pre-admission level of function 1 year post discharge from flu-associated hospitalization [2]. These prolonged negative outcomes result in a reliance of care support including family members, friends, and long-term care facilities. Further, declines in physical function, including frailty, are associated with increased mortality [3]. Thus, it is of the utmost importance that preventative measures, like vaccination, prevent severe flu infection and hospitalization in older adults to promote healthy aging and independent living.

### Challenges of current influenza vaccines

Seasonal influenza virus infects 5–15% of the human population yearly, causing about 500,000 deaths worldwide [4]. Influenza A and B commonly cause outbreaks in humans and acquire rapid mutations as they spread, reducing the potency of pre-existing immunity from previous infections or vaccination. Influenza A is further subtyped based on surface glycoproteins hemagglutinin (HA) and neuraminidase (NA), while influenza B is delineated by antigenic lineage. Point mutations to the surface HA and NA glycoproteins results in reassortment of gene segments across different strains in the same host [4]. Constant emergence and circulation of new strains makes yearly vaccination necessary. However, it is still often difficult to predict the strains that should be included in the year’s seasonal influenza vaccine formulation. Thus, the main limitations of current flu vaccines is strain specificity.

Current influenza vaccines typically contain four (quadrivalent) different strains. The World Health Organization (WHO) established the Global Influenza Surveillance and Response System. This is an organization that conducts year-round surveillance of influenza viruses, isolates circulating strains, and analyzes patterns of infections. For the majority of current flu vaccines, selected virus strains are propagated in embryonated chicken eggs and either inactivated or attenuated for vaccine production. The virus can adapt and mutate during this egg propagation and cause reduced protection against the intended circulating strain [5]. Since egg-based flu vaccine production to distribution takes about 6 to 8 months, there is ample time for mutations to occur, resulting in strain mismatch between the year’s vaccines and circulating strains [6]. This timeline makes it nearly impossible to quickly recreate the vaccine if different seasonal or pandemic influenza strains arise. Correspondingly, mismatches between circulating strains and vaccine strains are not uncommon. Overall, while influenza vaccines have been around for decades, their efficacy is quite variable for many reasons, including the age and health of the recipient, virulence of different seasonal strains, as well as the subtype and lineage of circulating viruses [7]. When the vaccine is well matched to circulating strains, the seasonal flu vaccine has a large range of efficacy with meta-analyses reporting a confidence interval of 46–74% vaccine efficacy for influenza A and 18–94% for influenza B [8]. Not surprisingly, many others have shown lower overall vaccine efficacy and wider ranges [9–12], however detailed reports of the closeness of the vaccine strain and predominant circulating strain is sometimes lacking in vaccine efficacy studies making it hard to interpret how effective flu vaccine is based on strain matching. In years when the vaccine is clearly not well matched, effectiveness is highly variable, ranging from 29 – 48% as seen between 2015 and 2019 [13] and 21–39% more recently in the 2019–2020 flu season due to antigenic drift [14]. Reported efficacy ranges also vary for the different subtypes with lowest efficacy generally in H3N2 compared to H1N1 and influenza B [9]. It is important to note that despite variable efficacy, influenza vaccination has been shown to prevent severe illness and potential complications from infections. It was estimated that vaccination prevented 7.1 million illnesses, 3.7 million medical visits, 109,000 hospitalizations, and 8,000 deaths from flu infections in the 2017 – 2018 flu season despite having an overall effectiveness around 38% [7, 15]. Importantly, despite these large ranges of efficacy based on circulating strains, flu vaccine efficacy is consistently reduced in older adults [11, 16]. Indeed, older adults have reduced overall vaccine efficacy, as well as lower antibody titers and reduced T cell proliferation [17]. Nonetheless, vaccination remains the best way to prevent severe flu infection, hospitalization, and associated complications, especially in older adults.

## Age-related declines in immunity that impact influenza vaccine responses

**Fig. 1:**
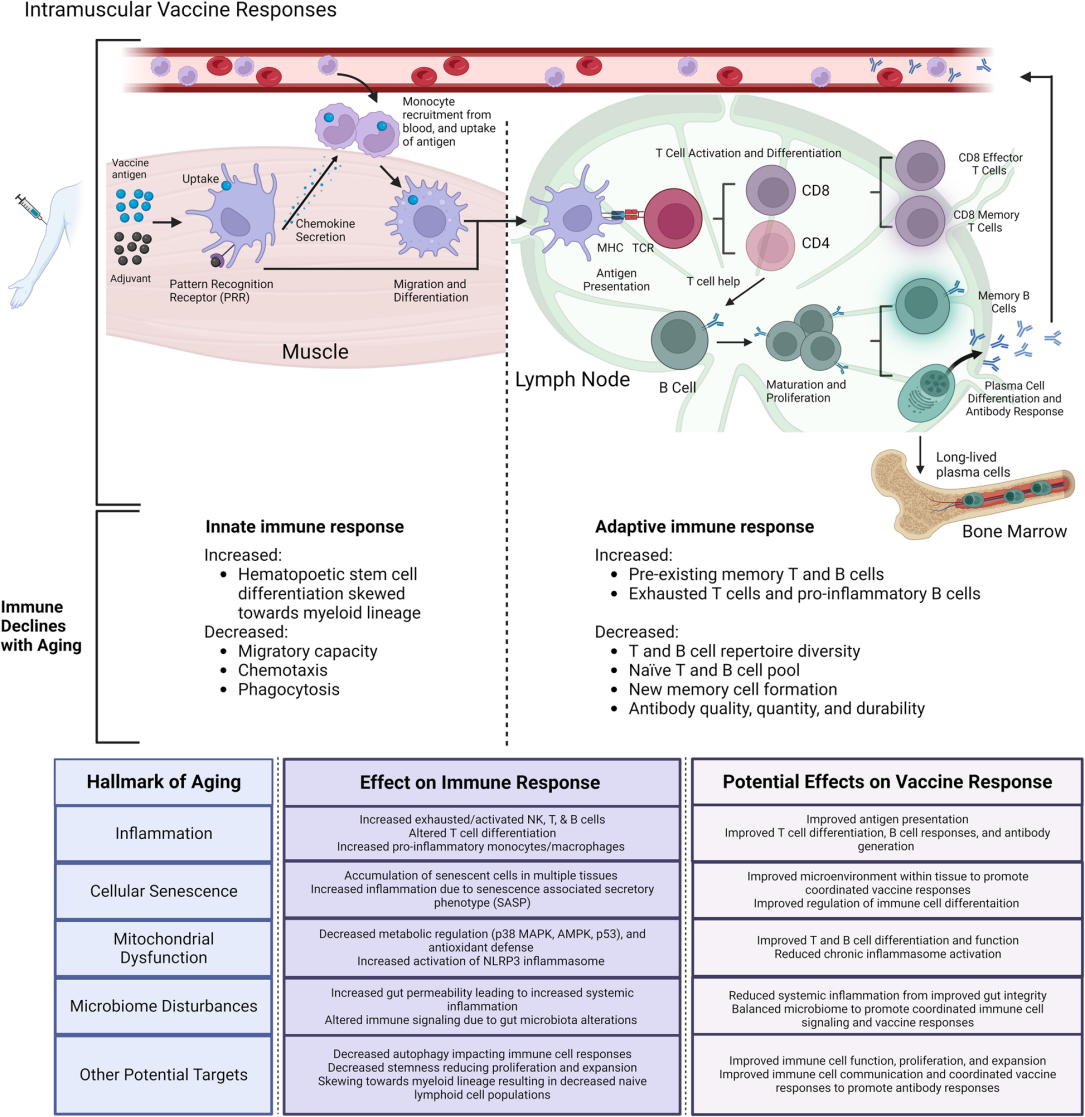
Immune response to vaccination and potential for targeting hallmarks of aging to improve age-related deficits in vaccine responses. Both innate and adaptive immune responses are induced by vaccination, which commonly occur via intramuscular injection. Adjuvants are sometimes included in vaccines to enhance immunogenicity. The vaccine is injected into muscle. Antigen is taken up by dendritic cells (DCs) and activate pattern recognition receptors (PRRs). DCs secrete chemokines that recruit other monocytes from the blood, where they migrate to the site of injection, and then uptake antigen and differentiate. Antigen experienced innate immune cells migrate to the lymph node and present antigen on Major Histocompatibility Complexes (MHC) to activate naïve T cells through T cell receptors (TCRs). Both CD4 and CD8 T cells can respond depending on the type of vaccination and MHC presentation. CD4 T cells will differentiate into various T helper subsets, where T follicular helper cells (Tfh) provide help to B cells. B cells in the lymph node can respond to both antigen and T cell help, which leads to maturation and proliferation into memory B cells and plasma cells. Short-lived plasma cells generate antibodies specific to vaccine antigen which circulate systemically. Long-lived plasma cells reside in the bone marrow and provide long-lasting antibody protection. These vaccine-induced cellular interactions are highly coordinated and depend on proper microenvironment milieu and specific immune cell functions and signaling. It is well known that various areas of the immune response to vaccination are impaired with aging as detailed in this review. We propose that targeting different hallmarks of aging can modulate the tightly coordinated responses to improve overall vaccine responses in older adults. Figure created with Biorender.com

The overall goal of vaccination is to induce protective immunity. It is important to understand how age-associated declines in immune responses lead to reduced protection from vaccines (See summary in Fig. 1). Elucidating the mechanisms underlying poor immunological protection in older adults from vaccination can result in more targeted interventions to improve vaccine responses, prevent infections, and reduce flu associated functional declines. Importantly, age-related declines in the immune system are not limited to one cell type, but rather affect multiple cells within the innate and adaptive immune system that are required for induction of optimal immunological protection. While the gold standard to assess vaccine responses is generally serum antibody titers, cell mediated responses are equally as important. Indeed, the evaluation of T cell responses to flu vaccination may be a more appropriate measure of protection in older adults [18–20]. T cell responses were more predictive of protection than antibody titers in older adults [20]. Further, ex vivo assays that determine interferon (IFN)-γ:interleukin(IL)-10 ratios as well as Granzyme B (GrzB) responses to flu vaccination were also more sensitive in determining risk for influenza illness after vaccination [20]. Unfortunately, with age, it has been shown that early upregulation of IFN-signaling pathways is impaired. In fact, early upregulation of IFN-signaling and antigen-presentation pathways after vaccination has been shown to be associated with higher influenza-specific antibody responses [21]. Importantly, certain vaccines that are received annually, such influenza vaccinations may also elicit poor innate immune responses due to pre-existing and cross-reactive antibodies that bind to vaccine antigen and limit the amount of antigen available for innate immune cell processing and presentation [22]. Thus, it is essential to understand age-related declines that are involved in innate responses, as well as both cell-mediated and humoral responses to vaccination.

### Age-related dysregulation in innate immunity that impact vaccine responses

Age-related changes in innate immune cells such as dendritic cells (DCs), neutrophils, macrophages, and Natural Killer (NK) cells contribute to poor vaccine responses [23–26]. Robust DC responses are crucial for effective uptake of antigens and trafficking to lymphoid organs, where they function as Antigen Presenting Cells (APCs). APCs are required for stimulating cell-mediated immunity to vaccination. These cells are responsible for providing three major signals to naïve T cells: 1) antigen recognition, 2) co-stimulation, and 3) cytokines [25]. The capacity of DCs to provide these signals to a sufficient level is known to decrease with age, thus impacting the ability to induce strong T cell activation [26]. These findings have been confirmed in mouse models where immunized aged mice show decreased peptide-MHC surface expression on APCs including DCs compared to adult mice [25]. Importantly, toll-like receptor (TLR) function in cell types including DCs and macrophages are also known to decline with age [27]. These receptors are essential pattern recognition receptors that trigger the expression of IFNs and proinflammatory cytokines. Importantly, in human aging, declines in TLR expression and function have been strongly associated with poor antibody responses to influenza vaccination [27].

Neutrophils and macrophages also experience age-related changes that affect their ability to communicate with other immune cell types and impair overall immune responses to vaccination. Neutrophils have reduced chemotaxis with age [28], causing poor directional movement toward a stimulus, such as towards the site of injection for vaccination. This is associated with a delay in neutrophil responses including degranulation, NETosis and recruitment of other immune cells [29]. Age-related declines in macrophage function also occur, including poor phagocytic ability and dysregulated transition between M1-like (pro-inflammatory) and M2-like (anti-inflammatory) phenotypes. As a result, impaired macrophage function with age results in poor inflammatory resolution, further influencing the ability of other immune cells to coordinate a response against an infection or generate immunological protection toward vaccination [29]. Recently, lymph node macrophages have been shown to play a role in initiating responses to influenza vaccination by secretion of various cytokines mainly IFN-β and IL-1α, where the latter promotes B cell responses in the lymph node [30]. Lymph node macrophages also recruit and activate NK cells following influenza vaccination via type I IFNs. Activated NK cells then produce IFN-γ which leads to the recruitment of IL-6 + CD11b + DCs. IL-6 production is essential for the development of humoral responses within the draining lymph node [31]. Thus, lymph node macrophages and NK cells play an integral role in promoting humoral responses to flu vaccination [31]. With age, NK cells have been shown to decrease in functionality with poor cytotoxicity as well as impairment in the secretion of cytokines and chemokines that affect other immune cell types [32]. In the context of influenza vaccination, increased NK cell activity is associated with better seroconversion in older adults [23], suggesting the early NK response is essential for humoral immunity. Additionally, it has been shown that with age, stimulated NK cells produce less IFN-γ, which can further impact DC maturation and T cell differentiation which are both essential for vaccine responses [32].

Although different innate immune cell types experience a wide variety of age-related dysfunction, these effects remain additive even though single deficits may seem manageable. Age-related changes to immune cell trafficking, TLR expression and signaling, as well as declines in antigen presentation have massively detrimental effects on the ability of the adaptive immune system to generate robust and coordinated responses. In totality, these various declines in functionality of different innate immune cell types leave older adults poorly protected from vaccination, and at a disproportionate risk for flu-related complications.

### Age-related dysfunction in cell-mediated immunity to vaccination

Due to age-related changes in innate immunity, the adaptive arm of the immune system begins at a disadvantage. Adaptive immune cell populations are equally susceptible to age-related declines and are known to have impaired function with aging. Thymic involution with age leads to overall decreased output of naïve CD4 and CD8 T cells [33]. Additionally, aged CD4 and CD8 T cells have dysregulated differentiation, function, and decreased T cell receptor (TCR) diversity among other deficits [34]. In response to flu vaccination, T follicular helper cells (Tfh) are essential for germinal center (GC) formation, B cell interactions, and robust antibody induction. In young adults circulating Tfh (cTfh) cells after vaccination are associated with increased antibody titers [35], however in older adults cTfh responses do not always correspond with antibody titers. For example, cTfh cells from older adults have increased basal expression of Inducible T-cell Costimulatory (ICOS), which is important for Tfh maintenance and Tfh-B cell interactions that result in differentiation into long lived memory cells and/or plasmablasts that produce high affinity antibodies [34, 36, 37]. In young adults, increases in ICOS expression on cTfh correlates with strong vaccine-induced IgM and IgG responses [36]. However, older adults fail to upregulate ICOS post vaccination [36] and there is impaired immunological protection post vaccination. Indeed, CD4 T cells have vast declines with aging that are both cell intrinsic and extrinsic [38]. In general, CD4 T cells have altered differentiation patterns with aging with a tendency to skew towards Th17 and altered Treg functionality among other changes in phenotype and function [38–41]. Regardless of phenotype, aged CD4 T cells have reduced IL-2 production following antigenic stimulation and impaired B cell help abilities leading to reduced humoral immunity [42–44]. Thus, age-related changes in CD4 T cell function directly contribute to impaired vaccine responses.

The generation of CD8 T cell immunity is important for providing protection from severe influenza infection. While current inactivated influenza vaccines on the market are not thought to induce potent CD8 T cell responses, CD8 T cell responses are desirable for durable cross-reactive protection. For example, vaccination with influenza virus-like particles including matrix (M1) that can induce CD8 T cell responses can protect mice from high-dose heterosubtypic influenza challenge [45]. Furthermore, T cell responses, such as GrzB induction and IFN-γ:IL-10 ratios, were more predictive of protection than antibody titers in older adults [20]. Thus, despite the limited CD8 T cell induction from current flu vaccines, CD8 T cell responses are still an important parameter for vaccine-induced immunity. Along with decreased naïve T cell and TCR repertoire diversity, CD8 T cells experience additional age-related declines, including reduced priming, proliferation, and quality of antigen-specific CD8 T cells [46], as well as overall impaired functionality [34, 47]. Similar to CD4 T cells, aged CD8 T cells also can take on a more suppressive phenotype [48]. Further, both the decline in repertoire diversity and a loss of reactivity to immunodominant epitopes have been correlated with impaired cellular immunity to de novo influenza infection as well as deficits in recall response to heterologous challenge [49]. Overall with aging, both primary and memory responses are impacted. Indeed, impaired CD8 T cell responses impact vaccine-induced immunity, as well as contribute to increased severity of flu infections in older adults.

### Age-related dysfunction in humoral immunity to vaccination

Age-related declines in adaptive immunity are not limited to T cell responses, but also impact B cells. Age-related declines in B cell responses to vaccination lead to reduced generation of high quality protective antibodies and contribute to the disproportionate impact of influenza infection on older adults [50]. There are reduced naïve B cells with age [50]. Importantly, with COVID-19, the number of circulating naïve B cells has been shown to have a strong association with antibody levels after vaccination [51]. Age-related declines in protective antibody responses to influenza infection also occur with fewer somatic hypermutations, which are point mutations which are crucial for affinity maturation where B cell receptors undergo selection in GCs of lymph nodes [9]. GCs are decreased in size and undergo declines in number with age likely due to poor Tfh-B cell interactions [43]. Along with reduced somatic hypermutations, there is also reduced class-switch recombination with aging, both likely mainly due to reduced activation-induced cytidine deaminase (AID) expression with aging [52]. In totality these changes contribute to various age-related deficits in B cell function including decreased quantity and specificity of antibodies, as well as reduced duration of protective immunity [50]. This further supports that declines in immune responses to vaccination in older adults is multifaceted. Together, these deficits in both the innate and adaptive immune system combine to result in impaired vaccination efficacy that leaves older adults more susceptibility to flu infection and at increased risk for severe infection. It is also important to recognize the phenomenon of original antigenic sin (OAS) in shaping humoral responses to vaccination in older adults. OAS explains that antibodies induced from primary exposure will continue to dominate regardless of subsequent challenges with similar, but slightly altered antigen. Since older adults likely have encountered influenza infection and/or have decades of annual flu vaccinations, their immune systems are already at a disadvantage in generating robust immunological protection to different strains in the seasonal vaccine. Thus far, there are no clear ways to alleviate the risk of OAS associated with annual influenza vaccination. However, it may be possible that adjuvants have the potential to induce cross-reactive memory B cells, which would be beneficial in improving immunological protection generated by vaccination [53].

It is clear from the preceding review that failure to generate sufficient immunological protection from vaccination in older adults is caused by a complex interplay of age-related changes in the global immunologic milieu (Fig. 1). Strategies that target singular deficits in the aged immune system may enhance some level of vaccine-induced immunity; however, it is not sufficient to improve overall protection to the levels seen in young adults since age-related immune deficits are not isolated. Nevertheless, to date, the focus of vaccine development has been reductionist and focused on very specific strategies that have evolved from vaccine science rather than geroscience, which can elucidate unique targets that can overcome a multitude of age-related deficits to improve vaccine efficacy in at-risk older adults.

## Current vaccination strategies to enhance protection in older adults

To overcome these deficits, specific vaccines were developed for older adults. Currently, a quadrivalent high dose flu vaccine (Fluzone) and adjuvanted flu vaccine (FLUAD) are FDA approved for older adults. The high dose vaccine contains four times more antigen than the standard dose vaccine. Clinically, it has been shown that the quadrivalent high dose flu vaccine is safe and well tolerated by older adults [54, 55]. Importantly, the high dose flu vaccine also showed increased seroconversion rates up to 28 days after vaccination, indicating that increasing the dose of antigen results improved immunogenicity and immunological protection [56]. Mechanistically, it has been shown that high dose flu vaccination induces a more potent Tfh response, which directly correlates to increased plasmablasts in older adults [57]. Although the exact molecular mechanisms behind improved vaccine responses to high dose flu vaccines are not completely defined, increasing the dose may alleviate certain deficits of the aged immune system, including poor antigen presentation by APCs [25]. In mouse studies, age-related changes in antigen presentation related components have been shown [25]. Additionally, preclinical work and clinical trials are underway to more closely evaluate the mechanisms of improved responses in both the innate and adaptive responses following high dose vaccination in older adults (NCT#:05154383). Rather than increasing the dose of antigen, the FLUAD vaccine utilizes an adjuvant, MF59. MF59 enhances immune cell recruitment to the site of injection via local chemokine and interleukin production, with CCR2 and ICAM-1 implicated as primary mediators [58, 59]. It also promotes immune cell activation, APC differentiation, and antigen presentation that further enhances CD4 T cell activation and GC reactions that directly influence B cell response increasing the magnitude and persistence of antibody responses to vaccination [58, 60]. More specifically, MF59 creates a more immunocompetent environment that works in a feed forward amplification loop where MF59 leads to increased chemokine production locally in the muscle tissue following injection, which then recruits more innate cells, mainly granulocytes and monocytes. These innate cells also respond to MF59 by increased chemokine secretion that recruits additional cells. The recruited cells take up adjuvant and antigen and transport to the draining lymph nodes to interact with antigen-specific T cells [59]. Indeed, there are increased APCs in the draining lymph node following vaccination with MF59 providing more opportunity for interaction with T cells [59]. Correspondingly, FLUAD enhances overall vaccine-induced protection in older adults [61–63]. While these vaccines consistently elicit more robust immune responses in older adults compared to the standard dose flu vaccine [64], protection is insufficient for preventing immense negative effects of influenza infection on older adults.

These current FDA-approved strategies to improve flu vaccine responses in older adults target specific deficits, mainly improving antigen presentation, cell trafficking to the site of injection, and improved antibody responses. Unfortunately, they are not able to address all age-related immune declines and protection is still inadequate which is evident since older adults continue to experience the most significant burden from flu infection, including 57% of hospitalization and over 75% of flu-related deaths [1]. Indeed, despite improved vaccine responses in older adults vaccinated with high-dose or adjuvanted flu vaccine compared to standard dose [64], efficacy rates are not high enough to prevent excess flu-associated hospitalizations and deaths in older adults. Further, the majority of currently approved flu vaccines still utilize egg-based technology that requires a lengthy manufacturing process. This poses a problem as the longer it takes for vaccine manufacturing, the more likely the that predicted circulating strains in the vaccine formulation could mutate causing a mismatch between the vaccine strain and predominant circulating strains, reducing overall efficacy for everyone. Alternative vaccination platforms that reduce vaccine production would be particularly valuable for all individuals. Furthermore, alternative vaccination approaches for older adults may be better suited to improve the tightly orchestrated immune response required for optimal vaccination responses. In fact, geroscience approaches that target common pathways of aging may be a better suited approach to alleviate multiple aspects of age-related declines and improve vaccine protection.

## Alternative vaccination platforms to enhance vaccination protection in older adults

Alternative vaccination strategies may be more fruitful to bolster responses in older adults. The COVID-19 pandemic has shed light on the benefits of mRNA-based vaccines. These benefits include a substantially shorter production time and high vaccine efficacy among older adults [65]. For example, it was reported that vaccine production only took about 2 months for the SARS-CoV-2 mRNA vaccine utilized for the first clinical trials (mRNA-1273) [65]. An important feature of mRNA-1273 vaccine development is the specific testing performed in older adults at different doses in early stages [66]. Early on, it was clear that older adults were disproportionately affected by COVID-19. Thus, including individuals in this age group for clinical trials provided unique biological context that is rarely considered in vaccine development. This study showed similar side effects between the lower and higher dose vaccine, and improved responses with the higher dose in older adults [66]. Importantly, Moderna’s mRNA-1273 vaccine showed 95.6% efficacy at preventing symptomatic SARS-CoV-2 infection in individuals 18 – 65 years old and those 65 years and older had 86.4% efficacy [67]. Similarly, the Pfizer-BioNTech BNT162b2 vaccine showed between 94–96% efficacy in all individuals from 16 to over 65 years old [68]. Although declines in vaccine efficacy are generally evident in older adults, the mRNA vaccine proved to have fairly consistent efficacy among different age groups. Studies have shown that mRNA vaccines were able to elicit similar responses in adults 70 years old and older as were seen in younger adults [69]. In adults over 80 years old, a striking 89% efficacy was noted, further suggesting that mRNA vaccination can elicit strong immunological protection in even the oldest cohorts of adults [70]. It is important to note, however, that the spike protein utilized to formulate this vaccine was a neoantigen for almost all humans, including older adults, and vaccine recipients likely had no pre-existing immunological memory. Thus, these results point to the ability of mRNA vaccines to overcome the breadth of immunological deficits in vaccine responses to new antigens, however it is not yet clear if this would also be the case for antigens that older adults have already encountered or been vaccinated for multiple times, such as influenza. Nonetheless, COVID-19 vaccine efficacy has been shown to have promising durability over time in both younger and older adults. Although risk of COVID-19 increases slightly after about 2 months post-vaccination, efficacy for all age groups plateaus at about 60–70% and remained at this level up to 8 months later [71]. It is likely that the inclusion of older adults in earlier clinical trial phases was important when selecting an appropriate dosage for this at-risk population [70]. Indeed, despite the widely accepted age-related reduction in vaccine responses, older adults remain underrepresented overall in clinical trials due to arbitrary upper age limits. In fact, 65.7% of trials on WHO Clinical Trials Registry Platform included at this time had an arbitrary upper age limit [72]. Ultimately, it is likely that many trials miss key age-related biological deficits that may help in understanding age-related requirements for eliciting stronger immunological protection from vaccination.

Several other factors contribute to increased efficacy with mRNA vaccines in older adults compared to other platforms. Specific to the SARS-CoV-2 virus, the mRNA platform allows generation of a more immunogenic spike protein than the wild-type virus due to prefusion-stabilizing mutations [73]. This ability to modify the structure combined with the potent lipid nanoparticle (LNP) delivery system is associated with prolonged protein expression, antigen specific Tfh induction, and GC B cell activation [74]. It’s possible that this mRNA approach could be applied to flu vaccine and allow for targeting of flu virus components that do not mutate as quickly, such as the stalk of HA, to provide better cross-reactive protection to different flu strains. Furthermore, these mRNA vaccines elicited both T helper 1-biased CD4 T cell responses as well as robust cytolytic CD8 T cell responses [65, 75, 76]. It is possible that the potent T cell responses stimulated by the mRNA vaccines contribute to the enhanced protection with age, since it has been shown that cell-mediated responses may be more indicative of protection in older adults [20]. While the evaluation is still in early stages, there seems to be overall improved efficacy utilizing mRNA vaccine platforms in older adults. Additional rigorous studies are needed to explore mechanisms of improved immunogenicity in this population.

Interestingly, many initial preclinical studies on mRNA-based vaccines were for influenza [77–80]. Similar to responses seen with the mRNA-based COVID-19 vaccines, mRNA-based flu vaccines showed robust responses including cTfh and memory B cells in nonhuman primates [78]. Importantly, studies have already shown that mRNA vaccines utilizing more conserved internal proteins, such as influenza nucleoprotein (NP), matrix protein 1 (M1), and LNPs increased survival after both homologous and heterosubtypic virus infection [79, 80]. Utilizing an mRNA vaccine for influenza also presents a unique opportunity to induce protection against multiple subtypes of the virus. Recent research has shown that a mRNA-based vaccine that contained 20 different HA mRNA-LNPs from all known influenza virus subtypes induces diverse antibodies in mice and ferrets that were successful in providing protection against both matched and mismatched viral strains, providing strong hope for a universal flu vaccine in the future [81]. Despite the preclinical success, the number of human trials investigating influenza mRNA vaccines is more limited likely owing to the rigorous FDA vaccine approval process and reduced necessity for rapid vaccine production prior to the COVID-19 pandemic. Furthermore, the trials to date focused on younger healthy adults. While these trials were successful and showed induction of protective antibody responses [82, 83], it is hard to predict if similar success would be evident in older adults. Comparisons between future mRNA-based flu vaccines and the current inactivated flu vaccines will be essential to determine if and how mRNA platforms can elicit stronger immune responses in individuals over 65.

While an mRNA vaccine platform may overcome many flu-specific vaccination challenges such as the production timeline, alternative strategies are likely still necessary to overcome the totality of age-related immune declines. Thus, a geroscience-focused adjunctive approach that targets common pathways of aging may be a more successful way to alleviate multiple aspects of age-related declines and improve overall flu vaccine protection in older adults.

## Targeting the hallmarks of aging to improve vaccine responses in older adults

The hallmarks of aging were established in 2013 [84]. Nine candidate hallmarks were identified that manifest during normal aging, accelerate aging when induced experimentally and improve age-related declines when reduced experimentally [84]. These hallmarks include mitochondrial dysfunction, cellular senescence, altered intracellular communication, and stem cell exhaustion among others [84]. Importantly, in 2022, 5 new proposed hallmarks were discussed among leaders in the aging research field including compromised autophagy, dysregulation in RNA splicing, inflammation, loss of cytoskeleton integrity and disturbance of the microbiome [85]. This list is likely to continue developing as aging research expands and we better understand the pathways and biology that contribute to age related declines. Thus, these hallmarks represent targets that may improve not only lifespan and healthspan, but also immunological protection from vaccination in older adults.

### Targeting hallmarks of aging: inflammation

Inflammation is crucial for generating immune responses by activating various signaling pathways. However, a chronic, sterile, pro-inflammatory state is associated with age and has recently been added to the hallmarks of aging. This phenomenon, also known as inflammaging [86], is likely driven by other hallmarks of aging including cellular senescence, mitochondrial dysfunction, and defective autophagy among others. Importantly, many studies have demonstrated that increased basal, systemic inflammation alters immune cell function and their ability to respond to challenges [87]. Indeed, pre-vaccination PBMC transcriptomic signatures that are more pro-inflammatory are negatively correlated with antibody responses, while signatures related to T and B cell function were positively associated with antibody responses [88]. Along these lines, inhibiting inflammatory cytokine production via pretreatment with an oral small molecule p38 mitogen-activated protein (MAP) kinase inhibitor was able to improve vaccine efficacy in older adults following subcutaneous varicella zoster virus (VZV) vaccination [89]. These findings may also translate to immune responses toward vaccines that are administered intramuscularly like flu vaccine. The JAK-STAT signaling pathway is a conserved pathway that results in the expression of a multitude of pro-inflammatory cytokines including IL-2, IL-6, IFNα and IFNβ [90], that are also commonly increased with aging [91]. Since the JAK-STAT pathway is implicated in autoimmune and inflammatory conditions, JAK-STAT inhibitors are FDA approved drugs used to treat various autoimmune, allergic, and inflammatory conditions [92]. Interestingly, JAK-STAT inhibitors decrease the senescence associated secretory phenotype (SASP), which is directly related to inflammaging in aged mice [93]. It is possible that utilizing JAK-STAT inhibitors as a pretreatment for vaccination may be able to reduce baseline levels of inflammation in older adults. However, targeting inflammation by inhibiting a major signaling pathway related to immune function comes with the risk of inhibiting the desired vaccination responses. Interestingly, it has been shown that older adults receiving tofacitinib, a common JAK-STAT inhibitor used to treat rheumatoid arthritis and ulcerative colitis, resulted in satisfactory, but not improved serological protection from flu vaccination compared to individuals in the age-matched placebo group (treatment group age range: 25–82 years old, placebo group mean age: 23–77 years old) [94]. On the other hand, a study analyzing the effects of similar drugs on SARS-CoV-2 vaccination responses indicated a reduction in antibody responses compared to placebo (treatment group mean age: 62.5 ± 11.8 years old, placebo group mean age: 64.3 ± 9.2 years old) [95]. Thus, more research is necessary to determine if JAK-STAT inhibition would improve flu vaccination responses in older adults. Interestingly, a small study showed that older adults who chronically use Non-Steroidal Anti-Inflammatory Drugs (NSAIDs) have higher titers of virus-neutralizing antibodies and increased antiviral defense gene expression after flu vaccination [96]. However, future studies need to replicate these findings in larger populations and determine mechanistic effects. On the other hand, the possibility that systemic inflammation has the potential to increase influenza vaccine immunogenicity has been proposed, although this has not been correlated with improved protection against disease [30]. Indeed, while some inflammation is necessary for optimal immune responses, excessive inflammation can impair responses, highlighting the highly coordinated immune signaling and microenvironment necessary for optimal vaccine responses. In totality, findings to-date suggest that increased basal inflammation has negative effects on vaccine-induced immune responses and antibody-mediated protection, and potentially could be targeted to improve vaccine responses. Overall, determining which players are the most appropriate and effective targets to interrogate the relationship between inflammaging and vaccine responses remains the biggest challenge as many pro-inflammatory signals are essential for proper vaccine responses as noted above. Mechanistic studies will be essential for determining the best targets for inhibiting baseline inflammation, without ablation of immune responses.

### Targeting hallmarks of aging: cellular senescence

Cellular senescence, as first described by Hayflick and Moorhead [97], is a mostly irreversible state of proliferation arrest that occurs as a response to cellular stress, including DNA damage and oxidative stress [98]. Senescent cells have been shown to serve important functions in embryonic development and wound healing in adult tissue and are readily cleared by the immune system after serving their functions [99, 100]. These cells, however, accumulate in much higher numbers with age and resist apoptosis, which causes systemic adverse effects. Senescent cells that resist clearance remain metabolically active and contribute to an inflammatory microenvironment. They secrete high levels of inflammatory cytokines, chemokines, and immune modulators, termed the senescence associated secretory phenotype or SASP, despite experiencing cell cycle arrest [101]. Some common SASP factor include IL-6, IL-8, and ICAM-1 among many others. SASP components can vary widely based on the inducer and duration of senescence, environment, and cell type [101]. Importantly, these SASP components also are major signals to immune cells and regulate many different functions [102]. In fact, SASP and the microenvironment of immune cells directly impacts their differentiation, activation, and cell–cell communication [103]. Clearing senescent cells via senolytics, a class of drugs that can induce apoptosis in senescent cells, induces various benefits in aged mice for age-related diseases such as liver cirrhosis [104], atherosclerosis [105], and type-2 diabetes [106]. However, our knowledge of the effects of senolytic drugs on immune responses is limited. To date, senolytics have been shown to alleviate senescence-induced changes in CD4 T helper (Th) subset differentiation in the context of flu infection [39] and reduce coronavirus induced-mortality in aged mice [107]. These findings may provide crucial insight into utilizing senolytics to re-balance differentiation patterns to reflect more youthful responses to primary infection. However, importantly, the effects of senolytics on vaccine responses have not yet been elucidated. Pre-vaccination treatment with senolytic drugs may be a unique geroscience approach to improve flu vaccine efficacy in older adults and would not require any novel vaccine development. Future research will elucidate the potential of senolytics to improve immunological resilience with aging.

### Targeting hallmarks of aging: mitochondrial dysfunction

Mitochondrial dysfunction is common with aging and has widespread impacts on both the systemic and cellular functions. Mammalian target of rapamycin (mTOR) and AMP-activated protein kinase (AMPK) are known as master regulators of metabolism. More recently the integral role of immune cell metabolism, also known as immunometabolism, on immune cell function has been elucidated [108]. Regulated metabolic pathways are essential for maintaining the overall health of cells including mobility, proliferation, and efficient utilization of nutrients to execute effector functions [108]. With aging, there is an overall increase accumulation of reactive oxygen species (ROS) and declines in mitochondrial activity within both tissues and individual cells [109]. Immune cells also experience mitochondrial dysfunction, which can contribute to different age-related dysfunctions in immunity [110]. mTOR inhibitors are at the forefront of metabolic targets being utilized to combat age-related mitochondrial dysfunction, including rapamycin derivatives (rapalogs) [111, 112]. Importantly, rapamycin and other mTOR inhibitors show similar effects in enhancing lifespan and improving aging-related diseases in mice [113, 114] and are currently being studied in various clinical trials. In fact, treatment with a rapalog in older adults prior to flu vaccination resulted in increased flu antibodies compared to those who received placebo [112, 115]. It is important to note that participants in this study discontinued treatment 2 weeks prior to flu vaccination. It is possible that this was done to prevent any subtle immune suppression that might interfere with vaccine responses, as rapamycin is used clinically (at much higher doses) as an immunosuppressant to reduce likelihood of organ transplant rejection. Although immune suppression seems counterintuitive when trying to elicit a strong immune response to vaccination, it is likely that mTOR inhibitors suppress the dysregulated activity that impairs immune responses with aging. Importantly, in addition to improved vaccination responses, older adults had reduced respiratory infections for 1 year following cessation of the mTOR inhibitor [115]. This finding suggesting that dysregulated mTOR activity contributes to poor immune responses overall and that targeting mTOR can improve overall immunological resilience in older adults. It is important to continue exploring various interventions that modulate cellular metabolism and determine the appropriate treatment schedules that maximize drug benefits while mitigating side effects and adverse events.

Metformin is FDA approved diabetes drug that modulates the AMPK/mTOR pathways among other metabolic targets and is also a candidate anti-aging drug [116]. Although traditionally used as a diabetes drug, metformin has shown positive effects on various immune cell types both through direct immunometabolic modulation and alleviation of inflammatory factors known to increase with age. Metformin treatment in vitro reduces age-related alterations in mitochondrial bioenergetics and inflammatory cytokine secretion in aged CD4 T cells [117]. Additionally, metformin treatment in young mice amplifies CD8 T cell memory formation [118], a known deficit in aged vaccine responses. In vitro B cell responses are also improved by metformin treatment in diabetics, including suppressing inflammatory mediators associated with poor antibody responses [119]. In vivo, post flu vaccine antibody titers were higher in metformin treated individuals compared to diabetics on other oral hypoglycemics [119]. Thus, metformin has clear utility in improving immune responses and cellular function in diabetics and it is possible this would translate to older adults. Metformin treatment also reduces circulating inflammatory markers including C-reactive protein (CRP), Interleukin-6 (IL-6) and Tumor Necrosis Factor-alpha (TNF- α) among other factors associated with conditions including frailty and chronic disease [120]. Thus, metformin targets multiple hallmarks of aging and may improve vaccine responses with aging.

In totality, rapamycin and metformin are two candidate drugs that may be able to target mitochondrial dysfunction with aging. Indeed, alternative drugs likely exist and/or can be developed that more specifically target age-related metabolic dysfunction. It is likely that targeting mitochondrial dysfunction in aging can improve vaccine responses, however future research should explore different drugs and potential interactions.

### Targeting hallmarks of aging: microbiome disturbances

The human microbiome includes various anatomical sites such as the skin, gastrointestinal tract, respiratory tract and urogenital tract among others. These various sites play a crucial roles in various physiological processes including metabolism, inflammation, and the immune system, making it a multifaceted target to address age-related declines. While targeting the skin, respiratory tract, or other microbiota sites may have utility for improving immune responses, the majority of research to date has focused on how the gut microbiome specifically can influence immune responses. Thus, we have focused this section on how the gut microbiota changes with age and can be a potential target to modulate immune responses in older adults. The gut microbiome shifts in composition due to many factors including age, diet, medications, and physical activity [121]. Further, leaky gut, or the increased permeability in the intestinal barrier, increases with age and allows for food antigens and bacteria among other factors to enter circulation and trigger immune responses [122]. Some have suggested a positive feedback loop where age-associated inflammation drives microbial dysbiosis as well [123]. As these mechanisms and pathways continue to be unveiled, targeting microbiome disturbances to treat aging may be an optimal target for improving immune responses to vaccination in older adults. Recent studies have shown that the use of probiotics, including lactobacillus species, successfully reduce gut permeability [124]. This improvement in intestinal barrier function would translate to decreases in leaky gut-induced inflammation and could potentially improve inflammation-induced immune response declines. In fact, the effects of various probiotic supplementations on flu vaccination response in older adults has been in various trials. Interestingly, three smaller trials explore supplementation with different probiotics including *Lactobacillus helveticus* R0052, *Lactobacillus rhamnosus* R0011 and fermented dairy drinks, all of which resulted in increased levels of flu-specific antibodies [125–127]. These smaller studies provide tremendous support for following up with larger studies to evaluate the ability of probiotics to improve flu vaccine responses with aging. It’s possible that probiotics are a safe and feasible intervention that can improve multiple hallmarks of aging via improving the microbiota and corresponding systemic inflammation.

### Targeting hallmarks of aging: other potential targets

It is important to remember that the hallmarks of aging are an incredibly interconnected web, each of which is sensitive to changes in other hallmarks of aging. In fact, the sheer heterogeneity of aging makes unveiling these connections quite difficult, but they remain crucial in our understanding of biological aging. Interestingly, the most often overlooked intervention to improve multiple age-related deficits, physical activity and exercise, also targets multiple hallmarks and has been shown to have profound effects on immune responses. Specifically, regular exercise has been shown to improve overall immunological function in terms of  responses to vaccination and infection, while also reducing the risk of various age-related diseases including type-2 diabetes and cardiovascular disease among others [128]. It is known that visceral adipose is increases with age, particularly in the abdominal region [129]. Importantly, increases in fat mass are also associated with increased incidence of metabolic dysfunction and inflammation. Thus, physical exercise can also reduce fat mass in older adults, which would also induce metabolic reprogramming and decrease systemic inflammation. In terms of the immune system, this is likely to have beneficial effects. Overall, studies have shown that exercise can improve overall immune function and vaccine responses in older adults. In older adults, moderate exercise can improve antibody titers to influenza vaccination [130]. Exercise immediately after vaccination also has utility to improve vaccine responses via myokines, mainly IL-6, improving CD4 T cell and B cell responses and increased type I IFNs that can improve DC activation and overall antibody responses. Indeed, it has been shown that 90 min of aerobic exercise immediately after immunization with either influenza or COVID-19 led to increases in antibody response several weeks later in adults [131]. This suggests exercise can improve vaccine responses regardless of vaccine platform as well. Although exercise has vast benefits in the overall health of older adults, including potentially improving immunological protection in response to vaccination, it may not be a viable option for those with extensive co-morbidities, disabilities, and/or uncontrolled medical conditions that may prohibit safe exercise. Thus, research on exercise mimetics may elucidate an alternative strategy to achieve similar exercise-induced benefits in vaccine responses as well.

Other hallmarks of aging aside from those detailed above also likely impact age-related immune deficits and represent potential targets. However, to date, different approaches to target these processes are limited and the direct link to declined vaccination responses has yet to be shown definitively. Stem cell exhaustion, for example, is a hallmark that directly impacts the immune system [132]. It is known that with age, the ability of stem cells to renew and to differentiate into other cell types is impacted [132]. Restoring the ability of stem cells to renew would theoretically reduce hematopoietic stem cell skewing towards myeloid cells and improve overall immune responses. Other hallmarks such as loss of proteostasis and reduced autophagy likely also have unique impacts on the immune system which require further exploration. Reduced autophagy likely contributes to impaired immune cell function due to the accumulation of damaged macromolecules and organelles [133], while loss of proteostasis leads to misfolded proteins that can be recognized by the immune system as foreign and contribute to sterile inflammation [134]. In totality, the interconnectedness of the hallmarks of aging and their effects on the entirety of the immune system likely have effects on vaccine efficacy and generation of protective immunity in older adults and should continue to be explored.

## Conclusions

Despite the availability of flu vaccines formulated to better protect older adults, older adults remain disproportionally at-risk for severe infection, flu-associated disability, and death. However, vaccination remains the most effective way to prevent infectious diseases and reduce severity of infections. Fortunately, the vast amount of research aimed to understand the hallmarks of aging have opened many doors to improve flu vaccine responses in individuals 65 years and older, potentially without the need to reformulate the vaccines themselves. Targeting aging as a whole, rather than specific age-related deficits, is likely more suited to improve the highly coordinated responses to vaccination and improve overall immunological resilience in older adults. The COVID-19 pandemic has shed light on the vulnerability of our older populations and the vast benefits that vaccination provides. It is important to acknowledge age-related immune changes as a hurdle that requires continued attention and investigation for future vaccine clinical trials. Alternative vaccine platforms for flu, such as mRNA-based vaccines, may be able to overcome some age-related immune deficits, while also providing improved production time and increased subtype inclusion to increase overall vaccine efficacy regardless of changes in predominantly circulating strains. Further, pre-vaccination treatments that target the hallmarks of aging may be a novel approach to improve flu vaccination responses with aging that don’t require any vaccine formulation changes. Overall, flu vaccine efficacy is integral to protecting older adults from excessive morbidity and mortality. Alternative vaccination strategies and pre-vaccination interventions that better address aging physiology likely can improve immunological resilience and overall protection in at-risk older adults.

## Data Availability

Data sharing is not applicable to this article as no datasets were generated or analyzed during the current study.
